# Investigating Potential Biomarkers in Autism Spectrum Disorder

**DOI:** 10.3389/fnint.2019.00031

**Published:** 2019-08-02

**Authors:** Carolyn Bridgemohan, David M. Cochran, Yamini J. Howe, Katherine Pawlowski, Andrew W. Zimmerman, George M. Anderson, Roula Choueiri, Laura Sices, Karen J. Miller, Monica Ultmann, Jessica Helt, Peter W. Forbes, Laura Farfel, Stephanie J. Brewster, Jean A. Frazier, Ann M. Neumeyer

**Affiliations:** ^1^Boston Children’s Hospital, Boston, MA, United States; ^2^Harvard Medical School, Boston, MA, United States; ^3^University of Massachusetts Memorial Medical Center, Worcester, MA, United States; ^4^University of Massachusetts Medical School, Worcester, MA, United States; ^5^Lurie Center for Autism, Massachusetts General Hospital for Children, Lexington, MA, United States; ^6^Child Study Center, Yale University School of Medicine, New Haven, CT, United States; ^7^Boston University Medical Center, Boston, MA, United States; ^8^Boston University School of Medicine, Boston, MA, United States; ^9^Center for Children with Special Needs, Floating Children’s Hospital at Tufts Medical Center, Boston, MA, United States; ^10^Tufts University School of Medicine, Boston, MA, United States; ^11^Autism Consortium at Harvard Medical School, Boston, MA, United States; ^12^Eunice Kennedy Shriver Center, University of Massachusetts Medical School, Worcester, MA, United States

**Keywords:** autism, ASD, biomarkers, serotonin, melatonin, dysmorphology, clinical research

## Abstract

**Background:**

Early identification and treatment of individuals with autism spectrum disorder (ASD) improves outcomes, but specific evidence needed to individualize treatment recommendations is lacking. Biomarkers that could be routinely measured within the clinical setting could potentially transform clinical care for patients with ASD. This demonstration project employed collection of biomarker data during regular autism specialty clinical visits and explored the relationship of biomarkers with clinical ASD symptoms.

**Methods:**

Eighty-three children with ASD, aged 5–10 years, completed a multi-site feasibility study integrating the collection of biochemical (blood serotonin, urine melatonin sulfate excretion) and clinical (head circumference, dysmorphology exam, digit ratio, cognitive and behavioral function) biomarkers during routine ASD clinic visits. Parents completed a demographic survey and the Aberrant Behavior Checklist-Community. Cognitive function was determined by record review. Data analysis utilized Wilcoxon two-sample tests and Spearman correlations.

**Results:**

Participants were 82% male, 63% White, 19% Hispanic, with a broad range of functioning. Group means indicated hyperserotonemia. In a single regression analysis adjusting for race and median household income, higher income was associated with higher levels of blood serotonin and urine melatonin sulfate excretion levels (*p* = 0.004 and *p* = 0.04, respectively). Melatonin correlated negatively with age (*p* = 0.048) and reported neurologic problems (*p* = 0.02). Dysmorphic status correlated with higher reported stereotyped behavior (*p* = 0.02) and inappropriate speech (*p* = 0.04).

**Conclusion:**

This demonstration project employed collection of multiple biomarkers, allowed for examination of associations between biochemical and clinical measures, and identified several findings that suggest direction for future studies. This clinical research model has promise for integrative biomarker research in individuals with complex, heterogeneous neurodevelopmental disorders such as ASD.

## Introduction

Autism spectrum disorder (ASD) is a highly heritable, heterogeneous neurodevelopmental disorder characterized by impaired social interaction and communication as well as restricted, repetitive behavior presenting in early childhood (American Psychiatric Association and DSM-5 Task Force, 2013). There is no curative treatment for ASD, but early intensive behavioral treatment can significantly improve long-term developmental outcomes (Lord and Mcgee, 2001; Estes et al., 2015; Reichow et al., 2018). However, specific measures needed to individualize treatment recommendations are lacking. Biomarkers could potentially identify clinically meaningful subgroups within highly heterogeneous populations and thus allow for more precise, individualized medical care by identifying risk, confirming diagnosis or guiding response to treatments (National Research Council, 2011; Veenstra-VanderWeele and Blakely, 2012; Insel, 2014; Interagency Autism CoordinatingCommittee [IACC], 2014; Ruggeri et al., 2014; De Los Reyes and Aldao, 2015; Varcin and Nelson, 2016). Recent authors have advocated for simultaneous measurement of multiple biomarkers to inform the understanding of the numerous systems and complex interactions that are likely to be involved in heterogeneous conditions (Hammock et al., 2012; Schendel et al., 2012; Ruggeri et al., 2014).

An extensive body of research is emerging on potential biomarkers in ASD including genetic, biochemical, proteomic, metabolomic, immune and redox markers as well as neuroimaging, electrophysiologic, physical and behavioral characteristics (Wang et al., 2011; Frustaci et al., 2012; Tordjman et al., 2013; Gabriele et al., 2014; Ruggeri et al., 2014; Varcin and Nelson, 2016).

Biochemical markers include neurotransmitters, hormones and markers of immune function and inflammation. Studies have consistently shown higher mean levels of platelet serotonin in individuals with ASD compared to controls (Schain and Freedman, 1961; Anderson et al., 1987; Cook and Leventhal, 1996; Mulder et al., 2004; Hammock et al., 2012; Gabriele et al., 2014; Pagan et al., 2014). Prior studies have also shown lower plasma levels of the pineal hormone melatonin and overnight urinary excretion of its major metabolite, melatonin sulfate, in individuals with ASD (Tordjman et al., 2005; Pagan et al., 2014). These biochemical markers can also vary with race, age, and gender (McBride et al., 1998; Duffy et al., 2011; Hammock et al., 2012). For example, elevations in platelet serotonin are particularly notable in prepubertal children with ASD (McBride et al., 1998).

A small number of studies have examined relationships between the chosen biomarkers and clinical characteristics in children with ASD. Elevated platelet serotonin levels have been associated in ASD with poorer speech development (Hranilovic et al., 2007), impaired social communication and play skills (Mulder et al., 2010), disruptive behavior (Kuperman et al., 1987), self-injury (Kolevzon et al., 2010), and higher autism severity (Abdulamir et al., 2018). Reduced urinary melatonin was associated with impaired social communication and play skills in ASD (Tordjman et al., 2012), and with higher serotonin levels (Mulder et al., 2010). Physical features such as macrocephaly, lower 2D:4D ratio and dysmorphic features are more common in individuals with ASD compared to controls and may correlate with symptom severity including lower IQ, language deficits and comorbid seizures (Courchesne et al., 1999, 2011; Manning et al., 2001; Miles et al., 2005; Sacco et al., 2007; Schaefer and Mendelsohn, 2008; Miller et al., 2010; Honekopp, 2012). Given the high rates of co-occurring conditions such as GI problems, sleep problems and seizures in ASD, studies examining the relationships of these conditions to biomarkers of interest could further inform understanding of subpopulations within the autism spectrum (Aldinger et al., 2015; Kohane, 2015).

Recently, studies have examined correlations between biomarkers or assessed more than one biomarker simultaneously (Sacco et al., 2010; Hammock et al., 2012; Schendel et al., 2012; Pagan et al., 2014; Ruggeri et al., 2014). For example, Hammock and colleagues found that oxytocin and serotonin were inversely related in individuals with ASD (Hammock et al., 2012). Pagan and colleagues examined associations of biomarkers with autism severity and clinical symptoms (e.g., melatonin with sleep disruption) and found that combined analysis of serotonin, *N*-acetylserotonin and melatonin levels in individuals differentiated individuals with autism from controls with 80% sensitivity and 85% specificity (Pagan et al., 2014). Sacco and colleagues collected information on a number of clinical traits in a study population and identified clusters of phenotypes (Sacco et al., 2010). The SEED study, a large national epidemiologic study of individuals with ASD and controls, is gathering data on multiple biomarkers and clinical characteristics (Schendel et al., 2012).

These types of studies can help elucidate potential etiologic pathways, distinguish cases from controls and identify subgroups of patients who may be phenotypically similar. One limitation of prior research, however, has been small sample sizes and participation bias. Identification of biomarkers that could be measured within the clinical setting would target large numbers of participants and potentially transform clinical care for patients with ASD (Hammock et al., 2012). Relatively few biomarkers, however, have been studied systematically in children with ASD, and the cost and feasibility of biomarker measurement varies. For example, neurophysiological biomarkers such as EEG require specialized equipment and staff training and cannot be easily completed during a regular follow-up clinic visit (Varcin and Nelson, 2016).

The clinical setting remains a relatively untapped resource for investigation of neurodevelopmental disorders including ASD for several reasons, among them differing priorities between clinical care and research, practical challenges including limited research infrastructure and cultural and attitudinal barriers in many clinical settings. Additionally, the specific core features of ASD such as sensory sensitivity and behavioral rigidity present barriers for individuals to participate in research.

To address these challenges, we conducted a multi-site demonstration project that evaluated the feasibility of integrating collection of biomarker and clinical data during ASD specialty clinic visits (Sices et al., 2017). Our primary hypotheses related to feasibility of the research model and we found that individuals with a range of developmental and behavioral functioning were able to participate and that the study activities did not interfere with clinical care. As part of the study, we collected multiple biomarkers on each participant to maximize the information collected in conjunction with a scheduled clinical visit (as opposed to a separate research visit). Our approach to assessment of correlations between biomarkers was exploratory. The chosen biomarkers, platelet serotonin and urinary melatonin sulfate, head circumference, dysmorphic status, and ratio of second and fourth digits (2D:4D), had prior evidence of association with ASD and could be measured with relative ease, efficiency and economy across multiple sites (Honekopp, 2012; Schendel et al., 2012; Teatero and Netley, 2013; Ruggeri et al., 2014; Mackus et al., 2017; Paynter et al., 2018).

This paper describes the range for platelet serotonin, urinary melatonin sulfate, head circumference, dysmorphic status and 2D:4D ratio in our clinical study population. In addition, this paper explores potential relationships among individual biomarkers, demographic features including sex, socioeconomic status (SES), and clinical features including cognitive level, behavioral function and co-morbid medical symptoms.

## Materials and Methods

### Participants

We recruited participants from five academic medical centers in Massachusetts that specialize in ASD assessment and treatment. Recruitment and classification procedures have been described previously (Sices et al., 2017). This study was approved by the Institutional Review Board at the lead site (Boston Children’s Hospital).

Children between 5 and 10 years old with a prior clinical diagnosis of ASD and scheduled for a regular clinic follow-up visit between April 2014–May 2015 at one of the five research sites were eligible to participate in the study. ASD diagnoses were verified by the children’s clinicians, all of whom were pediatric specialists with expertise in the diagnosis and treatment of ASD (developmental behavioral pediatricians, pediatric neurologists, nurse practitioners, and child psychiatrists). Participants’ ages were limited to between 5 and 10 years to improve stability of the ASD diagnosis and to increase the likelihood of pre-pubertal status, as some biomarkers vary with puberty.

Only one child per family was enrolled in the study. Exclusion criteria included having a non-English speaking caregiver or taking medication that could affect serotonin or melatonin metabolism within 2–6 weeks of the study visit (Supplementary Table 1). There were no exclusions for epilepsy, language impairment, level of intellectual functioning, or known genetic syndrome. Prior to conducting study activities, written informed consent was obtained from the participant’s legal guardian and informed assent was elicited from children age 7 years and older, who were able.

### Data/Sample Collection Measures

Parents/guardians completed a demographic and medical history form including birth history, medications, co-morbid medical symptoms, and family medical history. Parents/guardians also completed the Aberrant Behavior Checklist-Community (ABC-C), a 58-item behavioral functioning measure for children and adults with developmental disabilities that includes five subscales: irritability and agitation; lethargy and social withdrawal; stereotypic behavior; hyperactivity and non-compliance; and inappropriate speech (Aman et al., 1985). Scoring was completed using the method validated for children with ASD (Kaat et al., 2014).

Research staff reviewed medical records for each participant to identify results of the most recent cognitive testing (developmental or IQ test results). To allow for comparison amongst the various developmental and intellectual assessment measures documented across all participants, only non-verbal cognitive scores (Bayley cognitive score or non-verbal IQ) were analyzed. Study data were collected and managed using REDCap (Research Electronic Data Capture), a secure, web-based application for electronic data capture hosted at the lead site.

We estimated household income based on participant zip code as a proxy for SES. We assigned the median income reported in the 2012 U.S. Census for each participant’s zip code. Zip code regions were assigned to a quintile based on the national breakdown of income distribution (Geronimus and Bound, 1998; U.S. Department of Commerce, 2013).

As a part of each participant’s clinic visit, clinicians or their clinical staff obtained measurements of head circumference, height and weight. Clinicians then recorded results of a standardized dysmorphology examination (Miles et al., 2008; Sices et al., 2017). Photocopies of each participant’s hands were obtained by study staff following the clinic visit to provide 2D:4D ratios using established methodology (Manning et al., 1998). Photocopies that were not clear or did not demonstrate clear markings of the digits were omitted from analysis.

A 3 mL EDTA-anticoagulated whole blood sample was obtained from each participant for platelet count and serotonin analysis. Automated platelet count measurements were performed locally at each respective study site within 24 h of collection. Aliquots of whole blood were stored at −80°C until shipment on dry ice to the Anderson research laboratory at Yale University for platelet (whole blood) serotonin measurement, using previously published methodology (Anderson et al., 1987; Epperson et al., 2001). Overnight urine sample was collected for melatonin sulfate and creatinine analyses either from a first morning void, or an overnight diaper, thus representing nighttime production of melatonin for all samples. Urine samples were similarly stored at −80°C until shipment on dry ice to the Anderson laboratory. Melatonin sulfate-like immunoreactivity was measured by ELISA kit provided by IBL International (Toronto, ON, Canada) and creatinine levels were determined by HPLC using UV absorbance detection (240 nm) following 10-fold dilution (Hausen et al., 1981).

### Statistical Analysis and Considerations

Wilcoxon two-sample tests were used throughout to test for differences between groups of participants with and without a given characteristic. Spearman correlations were computed to estimate correlation between two continuous variables. Income was analyzed as a continuous variable. Race was challenging to analyze due to the small number of participants endorsing some racial categories, the number of participants selecting more than one category and the number selecting only the ‘Other’ category, which was not further specified. Linear regression models were used to test for effects of race and median household income on the two primary biomarker outcomes (platelet serotonin and urinary melatonin sulfate). A *p*-value < 0.05 was used as a cut-off for statistical significance. All tests were two-sided. Due to the exploratory nature of the study, no adjustments were made for multiple comparisons (Rothman, 1990).

## Results

A total of 88 participants were enrolled in the study; five were subsequently excluded from data analysis resulting in an analyzable sample of 83. Of the five excluded individuals, two did not provide at least one sample for biochemical measurement (blood for platelet serotonin or urine for melatonin sulfate excretion), two were discovered to be taking medications not disclosed at time of enrollment [fluoxetine (*n* = 1) and melatonin (*n* = 1)], and one voluntarily withdrew from the study.

### Group Descriptive Statistics

#### Demographics

Participant demographics are summarized in Table 1. The mean age of participants was 7.4 ± 1.6 years and 82% were male. Participants identified race and ethnicity by checking as many categories as applied. Overall, 63% endorsed White only, 7% Black only, 5% Asian only, 13% endorsed more than one racial category and 14% endorsed an “Other” category that was not further specified. Nineteen percent endorsed Hispanic ethnicity. Mean average income (estimated from zip code of residence) was $57,700 (SD 19,800), with a range from $26,944 to $121,693 across all participants (Table 1).

**TABLE 1:** Demographics and characteristics of study participants.

**Sex**	***n* (%)**
Female	15 (18%)
Male	68 (82%)

**Race°**	***n* (%)**

**Single Race**	72 (87%)
American Indian/Alaskan Native	0 (0%)
Asian	4 (5%)
Black/African American	6 (7%)
Other	10 (12%)
White/Caucasian	52 (63%)
**Multiple Races**	11 (13%)
Black and American Indian/Alaskan	1 (1%)
Black and White	5 (6%)
White and American Indian/Alaskan	1 (1%)
White and Asian	1 (1%)
White and Black and American Indian/Alaskan	1 (1%)
White and Other	2 (2%)

**Ethnicity**	**n (%)**

Hispanic	16 (19%)
Non-Hispanic	66 (80%)
Unknown	1 (1%)

**Age**	**Mean (SD)**

Age at diagnosis (years)	3.2 (1.6)
Age at visit (years)	7.4 (1.6)

**Income^∧^**	**Mean (SD), Median**

Income of participant’s zip code	$57.7K ($19.8K), $55.6

**Cognitive Function**	**N, Mean (SD)**

Non-verbal cognitive score	50, 88 (19)

***ABC-C***^#^**Checklist Subscale Score** **(n = 81)**	**Mean (SD)**

Irritability	13.5 (9.9)
Withdrawal	11.2 (7.8)
Stereotypy	5.5 (4.6)
Hyperactivity	15.3 (8.8)
Inappropriate Speech	4.7 (3.4)

**Genetic Testing**	**N (%)**

Had Genetic Testing Reported	58 (70%)
Known Genetic Syndrome	1 (%)
Variant of Unknown Significance	7 (%)

*^∗^Participants could endorse any applicable race; all responses tallied. ^#^Aberrant Behavior Checklist – Community. ^∧^Based on U.S. Census data from the 2012 Census.*

#### Developmental Function

ABC-C score distributions for the study group were comparable with recently published norms for children with ASD and represented a range of behavioral function (Kaat et al., 2014). Cognitive test results were available for 65 participants (78%). Mean age at cognitive testing was 5.4 years (*SD* = 2.2 years). Mean time elapsed from date of cognitive testing to date of biomarker sample collection was 2.3 years (*SD* = 1.8 years). Reported non-verbal standard scores ranged from 43 to 121 (mean = 88, *SD* = 19) with 14% having a non-verbal cognitive score below 70. Genetic testing results were reported for 58 patients. Of those, 1 had a genetic syndrome and 7 had a variant of unknown significance (Table 1).

#### Biomarkers

Discussion of study activity completion rates and feasibility has been previously described (Sices et al., 2017). Mean values and standard deviations as well as completion/collection rates for each of the biomarkers are shown in Table 2.

**TABLE 2:** Biomarker completion rates and group mean values.

**Measure**	***N* (%) completing measure**	**Mean (SD) or *N* (%) dysmorphic**
Serotonin (ng/mL)	73 (88%)	236 (92)
Platelet serotonin (ng/billion platelets)	71 (86%)	822 (407)
Urinary melatonin sulfate (ng/mg creatinine)	71 (86%)	131 (78)
Head circumference (z-score)	72 (87%)	0.77 (1.19)
2D:4D right hand	63 (76%)	0.96 (0.04)
2D:4D left hand	35 (42%)	0.96 (0.04)
Dysmorphology exam	75 (90%)	6 (8%)

#### Biochemical Markers

The serotonin and urinary melatonin sulfate excretion measures were non-normally distributed (Shapiro–Wilk tests, all *p*-values < 0.001). Mean levels for platelet serotonin in our participant group were elevated from previously published norms (McBride et al., 1998) consistent with the presence of hyperserotonemia. Urinary melatonin sulfate excretion rates in our ASD participant group, however, were not lower than prior reported rates in control populations (Tordjman et al., 2005) (Table 2).

Age was negatively correlated with melatonin sulfate excretion (*r* = −0.26, *p* = 0.048). We did not find an age association for serotonin. Contrary to previously published results, we did not identify a negative correlation between platelet serotonin and melatonin sulfate excretion levels (*r* = −0.008, *p* = 0.95). In addition, there were no correlations between platelet serotonin and melatonin sulfate excretion levels and sex, ethnicity, non-verbal cognitive level, or ABC-C subscale scores (Table 3).

**TABLE 3:** Biochemical markers by demographic characteristics.

**Patient characteristics**	**Platelet serotonin (ng/billion platelets) *n*, mean (SD)**	***p*-Value**	**Urinary melatonin sulfate excretion (ng/mg creatinine) *n*, mean (SD)**	***p-*Value**
**Sex**		0.10		0.20
Male	59, 852.5 (427.4)		58, 125.6 (77.9)	
Female	12, 671.0 (245.6)		13, 152.6 (76.1)	
**Ethnicity**		0.50		0.44
Hispanic	15, 732.9 (235.0)		14, 121.1 (72.2)	
Other	56, 845.6 (440.3)		56, 134.3 (79.6)	
**Non-verbal IQ**		0.28		0.29
Below 70	7, 1042.5 (657.2)		2, 144.0 (2.8)	
70 or above	38, 800.3 (401.4)		40, 131.5 (79.0)	

Due to the limited numbers of participants endorsing certain race categories we were not able to conduct comparative analyses. We examined platelet serotonin level and melatonin excretion rate by household income. The bivariate correlation of platelet serotonin and median household income was positive but did not reach statistical significance (*r* = 0.20, *p* = 0.09). In a single linear regression model testing for the effects both of race and of median household income on platelet serotonin, however, race and income were each independently associated with platelet serotonin. Household income was significantly associated with higher platelet serotonin levels in this regression with mean platelet serotonin increasing an estimated 9.8 points per $10K increase in household income (*p* = 0.004).

Melatonin sulfate and median household income were positively correlated (*r* = 0.25, *p* = 0.03) in bivariate analysis. In the linear regression model, higher median household income was associated with higher melatonin sulfate excretion levels with an estimated increase of 13.8 ng/mg creatinine per $10K increase in household income (*p* = 0.04).

### Physical Markers

2D:4D values were consistent with prior reports showing ratios for ASD participants below published norms (Manning et al., 2001). Head circumference values had a mean z-score 0.77 with range from −1.72 to 3.60. Twelve of the 72 participants (17%) with head circumference measurements were macrocephalic (z-score > 2SD). Dysmorphology examination was performed on 75 participants: 6 (8%) were identified as dysmorphic. Additionally, 42 of these participants (55%) had an abnormal finding in one or more body regions with the most frequently identified regions being the ear (20%), hair pattern (18%) and philtrum (16%). Dysmorphic status did not correlate with report of abnormal genetic test results. Only 3 of the 6 dysmorphic participants had cognitive testing results, scores ranged from 70 to 100 (Table 2).

### Co-occuring Conditions

Participants had high rates of caregiver reported co-occuring medical and psychiatric conditions including: 52% with gastrointestinal (GI) conditions, 40% with sleep problems, 41% with psychiatric conditions (including 25% with ADHD and 5% with anxiety disorder), 31% with history of regression in developmental skills, and 20% with neurologic conditions (including 7% with seizures) (Table 4).

**TABLE 4:** Caregiver-reported comorbid conditions.

**Condition**	***N* (%)**
**Neurodevelopmental/neurologic problems**	
Premature birth	11 (13%)
History of regression	26 (31%)
Neurologic conditions	17 (20%)
Seizures	6 (7%)
Headaches	2 (2%)
**Sleep problems**	
Any sleep problem	33 (40%)
Difficulty going to bed	15 (18%)
Difficulty falling asleep	19 (23%)
Unusually tired/sleepy during the day	13 (16%)
Difficulty waking up	13 (16%)
Very long or frequent naps	6 (7%)
Frequent or prolonged awakenings at night	17 (20%)
Sleepwalking or frequent nightmares	5 (6%)
No regular bed time and wake time	13 (16%)
**Gastrointestinal problems**	
Any GI condition	43 (52%)
Constipation	35 (42%)
Diarrhea	15 (18%)
Gastro-esophageal reflux disease (GERD)	10 (12%)
**Psychiatric and behavioral conditions**	
Any psychiatric disorder	34 (41%)
Anxiety disorder	4 (5%)
OCD	1 (1%)
ADHD	21 (25%)
Disruptive behavior disorder	6 (7%)
Depressive disorder	1 (1%)
Mood disorder	1 (1%)
Self-injurious	3 (4%)
Pica	2 (2%)
Enuresis or encopresis	2 (2%)

*Totals do not add up to 100% as some participants reported more than one condition in each category. No caregivers reported schizophrenia or psychosis.*

### Correlations of Biochemical Measures With Participant Characteristics

#### Biochemical Measures, Physical Characteristics, and Behavioral Functioning

Platelet serotonin levels and melatonin sulfate excretion were not significantly correlated with head circumference z-score, 2D:4D ratio, or dysmorphology status. There were also no associations among the physical measures (head circumference, 2D:4D, and dysmorphology status). Patients with dysmorphic status scored significantly higher than other patients on two of the ABC subscales [stereotypic behaviors: 10.0 (*SD*: 4.0) vs. 5.1 (*SD*: 4.5), *p* = 0.019; inappropriate speech 7.0 (*SD*: 2.9) vs. 4.5 (*SD*: 3.5), *p* = 0.043].

#### Biochemical Markers and Co-occurring Conditions

We examined correlations between both urinary melatonin sulfate excretion and platelet serotonin with co-occurring conditions including GI and neurologic problems, seizures, and sleep conditions reported by caregivers (Table 5). Patients with neurologic conditions (*n* = 15) had lower melatonin sulfate excretion than patients without these conditions [respectively: mean (SD): 91.4 (42.5) vs. 145.7 (81.3), *p* = 0.02]. There was no difference in mean age of participants with and without neurologic conditions (*p* = 0.69). No association was found between melatonin sulfate excretion and reported sleep problems (Table 5). We identified a trend toward an association between higher platelet serotonin levels and reported GI conditions (medians: 778 vs. 702, *p* = 0.08) Three participants had platelet serotonin values above 2000 ng/billion platelets (one was > 2 SD and the other two were > 3 SD above the mean); all three participants endorsed GI symptoms.

**TABLE 5:** Mean and standard deviation (SD) for biochemical markers in patients with co-morbid medical conditions, and comparisons between participants endorsing and not endorsing these conditions.

**Co-morbid conditions**	**Platelet serotonin (ng/billion platelets)**		**Urinary melatonin sulfate (ng/mg creatinine)**	
	***n*, Mean (SD)**	***p*-Value**	***n*, Mean (SD)**	***p*-Value**
**Gastrointestinal problem^∗^**		0.08		0.55
Yes	34, 934.3 (521.5)		36, 137.6 (86.4)	
No	37, 718.4 (222.5)		34, 120.9 (67.7)	
**Neurologic problem^∗^**		0.85		0.02^∗∗^
Yes	14, 858.8 (568.8)		15, 91.4 (42.5)	
No	52, 805.9 (372.5)		53, 145.7 (81.3)	
**Seizures^∗^**		0.85		0.12
Yes	5, 1068.2 (955.3)		4, 81.0 (46.7)	
No	57, 798.8 (359.4)		60, 141.0 (78.5)	
**Any sleep problem**		0.16		0.27
Yes	27, 761.4 (395.0)		29, 111.4 (61.0)	
No	44, 858.9 (414.0)		42, 143.7 (85.7)	

*^∗^Participants endorsing “not sure” for Gastrointestinal (*N* = 1) or Neurologic problem (*N* = 6), or “suspected” or “not sure” for Seizures (*N* = 10) excluded from analysis. ^∗∗^Significant at *p* < 0.05.*

## Discussion

In this pilot demonstration feasibility project, collection of multiple biomarkers during a regularly scheduled ASD specialty clinical visit allowed for the examination of associations between biochemical and clinical measures, and identified several findings that suggest direction for future studies. While our findings for individual biochemical and clinical biomarkers should not be viewed as definitive, we found associations between platelet serotonin and melatonin sulfate excretion with patient demographic and clinical characteristics that illustrate the potential of this approach to generate important information about multiple biomarkers and functional domains within a single heterogeneous clinical patient population.

Consistent with prior research, we identified elevated platelet serotonin levels in our sample, with means and ranges similar to previously published data for children with ASD (McBride et al., 1998; Mulder et al., 2004; Hammock et al., 2012). In contrast to previous studies, urinary melatonin sulfate excretion rates in our ASD participant group were not lower than prior reported rates in control populations (Tordjman et al., 2005). However, the limited available data on a similar age range and using a similar analytical methodology limit comparison of the melatonin sulfate results to prior reports. The overall racial distribution and the high proportion of participants endorsing more than one race precluded analysis of biomarker levels by race. Unexpectedly, however, we found that higher income, independent of race, was associated with higher platelet serotonin and higher melatonin sulfate excretion. The reasons for this are likely complex, as income estimated by zip code was used as a proxy for SES and may reflect multiple social and environmental factors including diet. Lower melatonin sulfate excretion was associated with increasing age during childhood in our sample as has been previously reported (Tordjman et al., 2005). In contrast, we did not find an age-based variation in platelet serotonin whereas prior studies found that serotonin levels correlate negatively with age in children with ASD (McBride et al., 1998; Hammock et al., 2012). This may be explained by the narrow age range of our study. A more recent study did not find an age-based difference in serotonin when comparing individuals with ASD who were below 16 years with those at or above 16 years (Pagan et al., 2014). Additionally, although a prior study showed a negative correlation between platelet serotonin and urinary melatonin (Mulder et al., 2010), the Pagan study did not find this correlation in a multi-aged (children and adults) sample of 230 patients with ASD (Pagan et al., 2014). Further studies with detailed sociodemographic variables will be needed to clarify these findings.

Frequencies of dysmorphic physical features in the study sample were similar to prior published findings for children with ASD. Dysmorphology examination identified six participants (8%) as dysmorphic using the scoring algorithm described by Miles et al. (2008). This compares to a rate of 12% reported as dysmorphic by Miles for a population of patients with autistic disorder. Of note, we did not preferentially select patients from settings that would typically have higher levels of dysmorphic features such as genetic specialty clinics. Although we did not have sufficient power to examine the relationship of dysmorphic status and cognitive functioning, dysmorphic status was correlated with higher ratings on the ABC stereotypy and inappropriate speech domains, suggesting higher symptom severity. This is consistent with prior studies documenting that patients with “syndromic autism” have more severe phenotypes (Miles et al., 2005; Schaefer and Mendelsohn, 2008).

Caregiver reported frequencies of comorbid medical and psychiatric conditions including developmental regression, sleep problems, ADHD, GI symptoms, and seizures were similar to those reported in other ASD cohorts (Richdale, 1999; Goldberg et al., 2003; Lord et al., 2004; Polimeni et al., 2005; Leyfer et al., 2006; Ibrahim et al., 2009; Buie et al., 2010; Murray, 2010; Aldinger et al., 2015). Study participants had lower levels of anxiety (5%) than previously reported (Filipek et al., 2000; Myers and Johnson, 2007; Volkmar et al., 2014), most likely explained by the study exclusion criteria that restricted participants on medications known to influence serotonin levels.

Serotonin [5-hydroxytryptamine (5-HT)] and melatonin are important in neurogenesis, synaptogenesis, mood, sleep, and GI function (Gabriele et al., 2014). Gastrointestinal symptoms were reported by 51% of participants, consistent with prior studies showing rates of GI symptoms ranging from 9 to 91% in children with ASD (Molloy and Manning-Courtney, 2003; Valicenti-McDermott et al., 2006; Ibrahim et al., 2009; Buie et al., 2010). A recent meta-analysis reported an odds ratio of 4.42 for GI problems in children with ASD (McElhanon et al., 2014). We did not identify significant associations between GI symptoms and platelet serotonin or melatonin sulfate excretion. However, we did observe a trend-level association between higher platelet serotonin and reported GI symptoms. In addition, three participants who were outliers with very high platelet serotonin (above 2000 ng/billion platelets) all reported GI symptoms. This is consistent with a recent study examining whole blood serotonin levels and co-occurring GI symptoms in 82 children age 6–15 years with ASD that identified a moderate positive correlation (*r* = 0.23, *p* = 0.048) between GI symptom score and serotonin levels in Caucasian participants (Marler et al., 2016). In addition, specific serotonin-related gene variants in individuals with ASD may contribute to gastrointestinal disturbance (Abdelrahman et al., 2014). Future studies examining serotonin levels in patients with ASD and co-occurring GI problems appear warranted.

Melatonin sulfate excretion was negatively associated with reported neurologic problems (*p* = 0.02), with much of the association due to lower levels in participants reporting diagnosed or suspected seizures. The rate of seizures (7%) was lower than that reported for other populations of children and adolescents with ASD. However, the age range of our study participants was between the two peak onset periods for seizures in ASD, early childhood and adolescence (Filipek et al., 2000; Myers and Johnson, 2007). An analysis of medical conditions in the AGRE and Simons Simplex Cohort (SSC) found similar rates of seizures (12.2 and 5.3% respectively) in two cohorts with mean age 9.2 and 9.0 years (Aldinger et al., 2015). Prior studies document lower melatonin secretion in individuals with refractory epilepsy compared to controls, and elevated melatonin levels following seizures; importantly, these studies did not examine associations with ASD (Bazil et al., 2000; Yalyn et al., 2006; Paprocka et al., 2010). Future studies examining urinary melatonin sulfate excretion levels in individuals with ASD and epilepsy would be of interest.

Variants in melatonin pathway genes have been associated with sleep onset delay (Veatch et al., 2015) and melatonin administration has been associated with improvements in sleep in children with ASD (Doyen et al., 2011; Guenole et al., 2011; Rossignol and Frye, 2011; Malow et al., 2012). Lower melatonin sulfate excretion levels were associated with reported insomnia in a population of 145 children and adults with ASD (Pagan et al., 2014). However, despite a high rate of reported sleep problems in our cohort (40%), there was no association found with melatonin sulfate excretion rates. This might be due to exclusion from study participation of individuals who were currently taking melatonin. Our rates of reported sleep problems were similar to those in unrestricted ASD populations (Sikora et al., 2012) but lower than those reported in the AGRE cohort (55.5%) and SSC (72.5%) (Aldinger et al., 2015).

Our study had several limitations. As this was a pilot demonstration study evaluating the feasibility of collecting data during specialty clinical visits, we did not have a control group. However, our primary intent was to examine whether and how individual biomarkers were inter-related within this clinical ASD population. Recruitment was solely from subspecialty tertiary care clinics. Our sample, however, was representative of the general population of individuals with ASD with regard to biomarker values and symptom severity. In addition, we view the direct integration of the research study within clinical visits as a strength that demonstrates the feasibility of the model.

The average income of our participants, as estimated by their home zip codes, is above the national average, and none of our participants were in the lowest national income quintile. However, the income ranges for participants are in line with the region surrounding all five clinical sites (U.S. Department of Commerce, 2013). We note that 11% of our participants endorsed more than one race. Given changing national demographics, it will become ever more important to consider how to best categorize and analyze data with respect to race and ethnicity.

We relied on expert clinician determination of ASD diagnosis, and parent report of behavioral function and comorbid medical and psychiatric symptoms. Cognitive data were historical, utilized a variety of measures, and were not concurrent with biomarker collection. In addition, availability of cognitive evaluation data, age at evaluation, and timing relative to this study varied across subjects (Sices et al., 2017).

Diet can affect both platelet serotonin and melatonin levels; however, dietary histories were not obtained. We also did not evaluate complete Tanner Staging or blood work to assess pubertal stage. Exclusion of individuals taking medications/supplements that could influence serotonin or melatonin levels likely reduced participation from some individuals with disruptive behavior, anxiety, and sleep problems.

The sample size, in combination with missing data for some variables, limited our power to identify smaller correlations. In the future, the use of clinical registries across multiple sites would facilitate systematic and consistent collection of data and tracking of outcomes for larger samples. Due to the exploratory nature of our study we did not correct for multiple comparisons, which increased the risk of Type I errors. Power was also impacted by the heterogeneity of the population sample. Future studies may need to look at subgroups of patients with particular symptom presentations or enriched samples to improve the ability to identify relationships.

This study examined potential correlations among biomarkers gathered during or in association with ASD specialty clinic visits. We found interesting potential correlations between biochemical markers, SES, and neurologic and GI symptoms that may be further explored in future studies. This translational research model has promise for defining subgroups of patients based on biomarker profiles and can help guide future studies on the use of biomarkers for individualized prognosis and treatment planning in individuals with complex, heterogeneous neurodevelopmental disorders such as ASD.

## Members of the Autism Consortium Biomarkers Study Clinicians Group

Boston University Medical Center: Marilyn Augustyn, Stephanie Blenner, Lynn Hironaka, Jennifer Radesky, Arathi Reddy, Jayna Schumacher, Laura Sices, Robert Keder Boston Children’s Hospital: Carolyn Bridgemohan, Elizabeth Harstad, April Levin, Leonard Rappaport, Alison Schonwald, Sarah Spence, Laura Weissman Center for Children With Special Needs, Floating Children’s Hospital at Tufts Medical Center: A. Stacie Colwell, Carmina Erdei, Eric Goepfert, Karen J. Miller, Christina Sakai, Monica Ultmann, L. Erik Von Hahn Lurie Center for Autism, Massachusetts General Hospital for Children: Jessica Helt, Yamini J. Howe, Ann M. Neumeyer, Christine Stine University of Massachusetts Memorial Medical Center: Roula Choueiri, David M. Cochran, Jean A. Frazier, Andrew W. Zimmerman.

## Data Availability

The datasets generated and/or analyzed during the current study are not publicly available due to inclusion of protected health information but are available from the corresponding author on reasonable request.

## Ethics Statement

This study was approved by the Institutional Review Board at the lead site (Boston Children’s Hospital): Assurance Identification No. FWA00002071 IRB Registration No. IRB00000352.

## Author Contributions

CB conceived of the study, participated in the design and coordination, analysis and interpretation of results and drafted the manuscript. DC, YH, LS, AZ, RC, SB, JF, and AN participated in the study design, coordination, analysis and interpretation of results and helped draft the manuscript. KM, MU, KP, LF, and JH participated in the coordination, analysis and interpretation of results and helped draft the manuscript. PF completed the statistical analysis, interpreted the results and helped draft the manuscript. GA participated in the design of the study, performance of biochemical measurements, interpretation of results and helped draft the manuscript. All authors read and approved the final manuscript.

## Conflict of Interest Statement

MA, RC, JF, AN, and KM were members of the Autism Consortium Steering Committee. The Autism Consortium was responsible for distributing the initial SFARI grant to the participating sites. SS receives other research funding from Simons Foundation Autism Research Initiative. SS received honoraria and travel support from Simons Foundation Autism Research Initiative in the past. GA has served as a consultant to Eli Lilly and Company and to Novartis Pharmaceuticals. JF has received research support from Fulcrum Therapeutics, Janssen Research and Development, and Roche as well as NICHD, NINDS, and NIMH. AZ has received fees for expert legal testimony and research support from the U.S. Department of Defense. The remaining authors declare that the research was conducted in the absence of any commercial or financial relationships that could be construed as a potential conflict of interest.
