# Supplementary material for: Investigating Potential Biomarkers in Autism Spectrum Disorder
Source: Front Integr Neurosci. 2019 Aug 2;13:31. doi: 10.3389/fnint.2019.00031 (PMC6687766; doi:10.3389/fnint.2019.00031)
Supplement: Supplementary file 1 [file Table_1.DOCX]

**Supplemental Table 1: Excluded Medications**

| **Excluded Medications** | **Washout Period** |
| --- | --- |
| Melatonin | 2 weeks prior to visit |
| Trazodone | 2 weeks prior to visit |
| Agomelatine | 2 weeks prior to visit |
| Ramelteon | 2 weeks prior to visit |
| SSRIs other than fluoxetine  (fluvoxamine, sertraline, citalopram, escitalopram, paroxetine) | 4 weeks prior to visit |
| SNRIs (atomoxetine) | 4 weeks prior to visit |
| Tricyclic Antidepressants (amitriptyline) | 4 weeks prior to visit |
| Monoamine Oxidase Inhibitors | 4 weeks prior to visit |
| Neuroleptics | 4 weeks prior to visit |
| Calcium Channel Blockers | 4 weeks prior to visit |
| Beta Blockers | 4 weeks prior to visit |
| Fluoxetine | 6 weeks prior to visit |
